# Molecular Epidemiology of Cross-Species *Giardia duodenalis* Transmission in Western Uganda

**DOI:** 10.1371/journal.pntd.0000683

**Published:** 2010-05-11

**Authors:** Amanda R. Johnston, Thomas R. Gillespie, Innocent B. Rwego, Traci L. Tranby McLachlan, Angela D. Kent, Tony L. Goldberg

**Affiliations:** 1 Center for Zoonoses and Infectious Disease Research and Department of Pathobiology, University of Illinois, Urbana, Illinois, United States of America; 2 Department of Environmental Studies and Global Health Institute, Emory University, Atlanta, Georgia, United States of America; 3 Department of Zoology, Makerere University, Kampala, Uganda; 4 Department of Natural Resources and Environmental Sciences, University of Illinois, Urbana, Illinois, United States of America; 5 Department of Pathobiological Sciences, Nelson Institute for Environmental Studies, University of Wisconsin, Madison, Wisconsin, United States of America; 6 Center for Global Health, University of Wisconsin, Madison, Wisconsin, United States of America; Liverpool School of Tropical Medicine, United Kingdom

## Abstract

**Background:**

*Giardia duodenalis* is prevalent in tropical settings where diverse opportunities exist for transmission between people and animals. We conducted a cross-sectional study of *G. duodenalis* in people, livestock, and wild primates near Kibale National Park, Uganda, where human-livestock-wildlife interaction is high due to habitat disturbance. Our goal was to infer the cross-species transmission potential of *G. duodenalis* using molecular methods and to investigate clinical consequences of infection.

**Methodology/Principal Findings:**

Real-time PCR on DNA extracted from fecal samples revealed a combined prevalence of *G. duodenalis* in people from three villages of 44/108 (40.7%), with prevalence reaching 67.5% in one village. Prevalence rates in livestock and primates were 12.4% and 11.1%, respectively. Age was associated with *G. duodenalis* infection in people (higher prevalence in individuals ≤15 years) and livestock (higher prevalence in subadult versus adult animals), but other potential risk factors in people (gender, contact with domestic animals, working in fields, working in forests, source of drinking water, and medication use) were not. *G. duodenalis* infection was not associated with gastrointestinal symptoms in people, nor was clinical disease noted in livestock or primates. Sequence analysis of four *G. duodenalis* genes identified assemblage AII in humans, assemblage BIV in humans and endangered red colobus monkeys, and assemblage E in livestock and red colobus, representing the first documentation of assemblage E in a non-human primate. In addition, genetic relationships within the BIV assemblage revealed sub-clades of identical *G. duodenalis* sequences from humans and red colobus.

**Conclusions/Significance:**

Our finding of *G. duodenalis* in people and primates (assemblage BIV) and livestock and primates (assemblage E) underscores that cross-species transmission of multiple *G. duodenalis* assemblages may occur in locations such as western Uganda where people, livestock, and primates overlap in their use of habitat. Our data also demonstrate a high but locally variable prevalence of *G. duodenalis* in people from western Uganda, but little evidence of associated clinical disease. Reverse zoonotic *G. duodenalis* transmission may be particularly frequent in tropical settings where anthropogenic habitat disturbance forces people and livestock to interact at high rates with wildlife, and this could have negative consequences for wildlife conservation.

## Author Summary

*Giardia duodenalis* is a common protozoan parasite that infects multiple mammalian species, including humans. We analyzed *G. duodenalis* from people, livestock, and wild non-human primates in forest fragments near Kibale National Park, western Uganda, where habitat disturbance and human-animal interaction are high. Molecular analyses indicated that endangered red colobus monkeys were infected with *G. duodenalis* assemblages BIV and E, which characteristically infect humans and livestock, respectively. *G. duodenalis* infected people at rates of up to 67.5% in one village, and people age 15 years or younger were especially likely to be infected. *G. duodenalis* infection in people was not associated with other factors related to behavior and hygiene, and infected people were no more likely to have reported gastrointestinal symptoms than were uninfected people. These results demonstrate that *G. duodenalis* transmission from humans and domestic animals to wildlife may occur with ease in locations such as western Uganda, where habitat disturbance causes ecological overlap among people, livestock, and primates. This conclusion has conservation implications for wildlife such as red colobus, which are already endangered by habitat loss.

## Introduction

*Giardia* is a genus of parasitic protozoan that infects the small and large intestines of a broad range of vertebrate hosts [1]. Considered among the most common human intestinal protozoa, especially in the tropics [2], *G. duodenalis* ranges in clinical severity from asymptomatic to highly pathogenic [3]. Both host factors (e.g. nutrition, immunity, co-infection with other agents) and pathogen factors (e.g. strain, infectious dose) are thought to contribute to the clinical severity of giardiasis [2].

*G. duodenalis* is also notable for cross-species transmission, including zoonotic transmission [3], [4]. Molecular techniques have shed considerable light on this aspect of *G. duodenalis* ecology [5]. Sequencing of phylogenetically informative genes has, for example, revealed transmission among humans, dogs (*Canis familiaris*), and cattle (*Bos taurus*) in Italy [6], and among humans, cattle and mountain gorillas (*Gorilla beringei beringei*) in Uganda [7]. At present, six *G. duodenalis* “assemblages” (A–G) are recognized, infecting a range of mammalian hosts and likely representing as many distinct species [8]. Most studies that have applied molecular methods to *G. duodenalis* in wild mammals have found that samples fall into assemblages A or B, which characteristically infect people, concluding that the animal hosts involved may represent reservoirs of infection for humans (e.g [7], [9]–[13]).

We conducted a cross-sectional study of *G. duodenalis* in rural western Uganda, near Kibale National Park, a location of high human-livestock-wildlife overlap and conflict [14]. We sampled people, livestock, and wild non-human primates associated with forest fragments outside of the protected areas of the park, where primates interact frequently and often antagonistically with people [14], where we have documented increased rates of human-primate-livestock bacterial transmission [15], and where we previously documented a *G. duodenalis* prevalence of 3.8% in primates using microscopy [16]. Our goal was to use molecular methods to assess the prevalence and cross-species transmission potential of *G. duodenalis* in this area, and to examine risk factors for infection and clinical disease.

## Materials and Methods

### Ethics statement

Prior to data collection, all protocols were reviewed and approved by the Uganda National Council for Science and Technology and the Uganda Wildlife Authority, as well as by the Institutional Review Board of the University of Illinois and the Animal Care and Use Committee of the University of Illinois. Due to low literacy rates, oral informed consent was obtained by trained local field assistants and documented by witnessed notation on IRB-approved study enrollment forms.

### Study site

The study took place in Kibale National Park, western Uganda (0°13′–0°41′N, 30°19′–30°32′E), and in surrounding forest fragments. This habitat consists primarily of moist semi-deciduous and evergreen forest, between approximately 1,100 and 1,600 m in elevation [17], [18]. Kibale supports an exceptionally high species diversity and density of primates [19]. The Kibale region is noted for its rapidly expanding human population and intensive human-wildlife ecological interaction and conflict [14]. This conflict is especially pronounced outside of the protected areas of Kibale, in remnant forest fragments that sustain small populations of primates [20].

For the present study, we focused on three such forest fragments, Bugembe, Kiko 1, and Rurama, and the villages surrounding them. These sites, which are described in detail elsewhere [15], range from approximately 0.7 to 1.5 km^2^ and contain small populations of monkeys (between 4 and 60 individuals of up to three species; see below). Our previous investigations have shown that rates of human-primate-livestock bacterial transmission are elevated in these locations due to anthropogenic habitat disturbance related to timber harvesting, agriculture, and the collection of forest products (e.g. firewood) [15]. Primates inhabiting these fragments also have elevated prevalence of infection with parasitic nematodes and protozoa, including *G. duodenalis* [16], [21].

### Sample collection and surveys

In May and June, 2007 (dry season), we collected fecal samples from two folivorous monkey species, the red colobus (*Procolobus badius tephrosceles*; *n* = 30) and the black-and-white colobus (*Colobus guereza*; *n* = 29), and one omnivorous monkey species, the red-tailed guenon (*Cercopithecus ascanius*; *n* = 22), from undisturbed areas within Kibale National Park, as well as from the highly disturbed Bugembe, Kiko 1, and Rurama forest fragments. We collected single fecal samples non-invasively from individual animals on 13 days, at the same time that we collected demographic information and behavioral observations, as previously described [15].

We sampled local human volunteers (*n* = 108) and their livestock (cattle, *Bos taurus* and *B. indicus*, *n* = 25; goats, *Caprus hircus*, *n* = 57; and sheep, *Ovis aries*, *n* = 7) in villages surrounding each forest fragment. People and livestock in these villages use forest fragments intensively for such purposes as firewood collection and grazing, respectively, thus increasing ecological overlap with primates [14]. People were selected from among willing volunteers within target households, which were chosen at random from households within 0.5 km from the forest fragment edge [15]. This protocol yielded multiple samples from the same households, but individual volunteers were sampled only once. Volunteers were instructed in the proper method for placing fecal samples into sterile cups, which were collected the following day.

Livestock were sampled from households where human volunteers resided. As with people, multiple samples were collected from the same households, but individual animals were sampled only once. Fresh livestock feces were collected from the rectum using a sterile glove, or from the ground if the animal was observed to defecate, with care taken to avoid environmental contamination by sampling only those portions of the fecal material that had not contacted the ground. Fecal samples were transported to our field laboratory as soon as possible after collection (within 6 hours). 1 ml of fecal material from each sample was homogenized with an equal volume of RNA-later nucleic acid stabilizing buffer (Ambion) and stored at −20°C in the field prior to transport to the United States.

Concurrent with human fecal sample collection, a survey was administered to each participant. The survey focused on demography, gastrointestinal symptoms, patterns of land use, and interactions with animals during the four-week period prior to sample collection. The survey was administered in Rutooro (the local language) by trained field assistants who were also members of the local communities; response biases caused by the presence of foreigners were thereby avoided.

### Molecular methods

DNA was extracted from 500 µl of fecal+RNA-later suspension using the FastDNA Spin Kit for Soil (MP Biomedicals, Solon, OH), followed by an additional wash and ethanol precipitation in the presence of CTAB in order to remove potential PCR inhibitors [22]. Samples were tested for the presence of *G. duodenalis* DNA using a real-time PCR targeting the small subunit RNA gene on an ABI Prism® 7000 Sequence Detection System (Applied Biosystems, Inc.), with primers, probe, and cycling conditions following published methods [23]. Samples were run in duplicate, using 1 µl of extracted DNA template per reaction.

To infer genetic relationships among *G. duodenalis* from samples that tested positive by real-time PCR, we conducted conventional PCR amplification and direct sequencing of four *G. duodenalis* genes. Conditions were optimized for each locus separately using the Failsafe PCR System (Epicentre Biotechnologies, Madison, WI) according to the manufacturer's recommendations and based on published primers and protocols for each gene: *ef1-α* [24], *gdh* [25], SSU-rDNA [26], [27], and *tpi* [24] (complete protocols are available upon request).

PCR products were electrophoresed on 2.0% agarose gels containing 0.5µg/ml ethidium bromide in 1× Tris-acetate-EDTA buffer at 100 volts for 30–45 minutes at room temperature (sub-cell model 192 electrophoresis system, Bio-Rad, Hercules, CA). Each well contained 25 µl of PCR product and 4µl 6× loading dye or 6 µl of 0.5 mg/ml GeneRuler 100-bp DNA ladder (Fermentas, Glen Burnie, MD). Amplicons were visualized under UV light, and bands of the predicted sizes were excised for DNA extraction using the Zymoclean Gel DNA Recovery Kit (Zymo Research, Orange, CA), according to the manufacturer's protocol.

Following extraction, amplicons were sequenced in both directions on ABI 3730XL capillary sequencers located in the Roy J. Carver Biotechnology Center at the University of Illinois at Urbana-Champaign. Sequences were edited using the computer program Sequencher, Version 4.2 (Gene Codes Corporation, Ann Arbor, MI) and were queried against known sequences using BLAST [28]. Population genetic and phylogenetic analyses were conducted using the computer program MEGA4 [29].

## Results

Real-time PCR detected *G. duodenalis* in 64 (23%) of 278 total fecal samples (Table 1). Overall, the prevalence of *G. duodenalis* was higher in humans than in either non-human primates (“primates” hereafter) or livestock (Table 1). In humans, the prevalence of *G. duodenalis* was markedly higher in Rurama community than in either Bugembe or Kiko 1 communities (Table 1), and this difference was statistically significant (odds ratio (OR) = 6.2; 95% Wald confidence limits 2.6–14.7; Fisher's exact *P*<0.001). No similar differences were observed in the prevalence of *G. duodenalis* in livestock among these locations, however (Table 1).

**Table 1:** Prevalence of *Giardia duodenalis* in humans, livestock, and primates in and near Kibale National Park, western Uganda.

	Humans		Livestock		Primates	
Location	*n*	Prevalence^a^	*n*	Prevalence^a^	*n*	Prevalence^a^
Bugembe	32	28.1 (15.4–45.5)	26	19.2 (8.1–38.3)	23	26.1 (12.3–46.8)
Kiko 1	36	22.2 (11.5–38.3)	37	8.1 (2.1–22.0)	–	–
Rurama	40	67.5 (51.9–80.0)	26	11.5% (3.2–29.8)	30	6.7 (0.8–22.4)
Kibale	–	–	–	–	28	3.6 (0–19.2)
All	108	40.7 (31.9–50.2)	89	12.4% (6.9–21.0)	81	11.1 (5.8–20.0)

a Prevalence rates are percentages of individuals positive by real-time PCR; numbers in parentheses indicate 95% confidence intervals calculated according to the modified Wald method [45]. Humans and livestock do not inhabit Kibale National Park, and non-human primates have been extirpated from Kiko 1 forest fragment [14], so prevalence data are not available for these species/locations.

The prevalence of *G. duodenalis* in humans was higher in individuals 15 years or younger (n = 62; 53.2%) than in individuals between 16 and 75 years (n = 45; 22.2%), and this difference was statistically significant (OR = 4.1; 95% Wald confidence limits 1.7–9.7; Fisher's exact *P*<0.001). Subadult livestock (n = 24) harbored *G. duodenalis* at a higher rate (25.0%) than did adult livestock (n = 65; 7.7%), and this difference was also statistically significant (OR = 2.7; 95% Wald confidence limits 1.1–14.7; Fisher's exact *P* = 0.037), mirroring the trend in humans. We were unable to examine age as a risk factor for *G. duodenalis* infection in primates, due to the difficulty of determining the ages of individual wild primates.

Data from survey responses (83.5% of people responding) were used to identify risk factors for human *G. duodenalis* infection related to land use, demography, and behavior (Table 2). No factors related to behavior were significantly associated with infection status, including working in fields, working in the forest, fetching water from an open water source (e.g. a pond or stream), fetching water from a protected well, or tending livestock (Table 2). Residence in a household with at least one other *G. duodenalis*-positive person was significantly associated with infection, as was residence in a household with at least one positive cattle, goat, or sheep.

**Table 2:** Univariate analyses of risk factors for infection with *Giardia duodenalis* in people near Kibale National Park, western Uganda.

Variable^a^	*n* b	OR	95% CI^c^		*P* d
			Lower	Upper	
**Age (≤15 years)**	108	4.10	1.74	9.70	<0.001
Sex (male vs. female)	107	1.08	0.50	2.34	0.500
**Location (Rurama vs. Kiko 1 or Bugembe)**	108	6.23	2.64	14.72	<0.001
Worked in agricultural fields	90	0.91	0.35	2.39	0.673
Worked in the forest (e.g. collected firewood)	90	1.16	0.45	3.00	0.476
Fetched water from an open water source	90	0.45	0.19	1.10	0.063
Fetched water from a closed well	90	0.86	0.35	2.09	0.458
Tended livestock	90	0.99	0.38	2.58	0.609
Lived in household with another positive person	107	3.46	1.27	9.43	0.010
Lived in household with positive livestock	108	2.49	1.01	6.13	0.037
Experienced gastrointestinal symptoms	86	1.23	0.51	2.98	0.411
Used traditional or commercial medicines	89	0.90	0.34	2.41	0.519

a Variables based on survey responses refer to the four-week period prior to sample collection. Variables in bold were retained in a final multiple logistic regression model (see text).

b Numbers of observations differ among variables due to incomplete reporting of information on some surveys.

c Wald 95% confidence intervals around odds ratios (OR).

d *P* values were calculated using Fisher's exact tests.

We found no association between infection with *G. duodenalis* and the reporting of gastrointestinal symptoms or the taking of medicines (Table 2). This was despite a strong association between the reporting of gastrointestinal symptoms and the reporting of medication usage during the same time period (OR = 48.3; 95% Wald confidence limits 6.1–383.4; Fisher's exact *P* = 0.001). The lack of an association between infection status and the reporting of gastrointestinal symptoms held true even when individuals ≤15 years (n = 48) were analyzed separately (OR = 1.15; 95% Wald confidence limits 0.37–3.64; Fisher's exact *P* = 0.704).

To account for potential confounding effects, we used multiple logistic regression with various strategies of model selection (e.g. forward addition, backward stepwise elimination; details not presented). Regardless of the method used, the same two variables were retained in the final model in all cases: age of ≤15 years (aOR = 4.78; Wald 95% CI = 1.79–12.78; chi-square = 9.76; *P* = 0.002) and residence in Rurama (aOR = 5.40; Wald 95% CI = 1.88–15.50; chi-square = 9.83; *P* = 0.002). Residence in a household with another positive person or residence in a household with a positive animal, which were significant in our univariate analysis, fell out of the model as non-significant when all predictors were considered together.

All sequences generated during this study were submitted to GenBank (Accession Numbers GQ502935–GQ503034). Our success in generating *G. duodenalis* nucleotide sequences from the 64 positive samples identified by real-time PCR varied by locus, with success being highest for SSU-rDNA (Table 3). Genetic diversity also varied considerably among the *G. duodenalis* loci, with *tpi* containing the highest overall nucleotide diversity and *ef1-α* the lowest (Table 3). Genetic diversity was marginally higher among *G. duodenalis* from humans than from other species at three of four loci (Table 3).

**Table 3:** Nucleotide-level genetic diversity at four loci in *Giardia duodenalis* from people, primates, and livestock near Kibale National Park, Western Uganda.

Population	*ef1-α* (147 bp)		*gdh* (393 bp)		SSU-rDNA (151 bp)		*tpi* (225 bp)	
	n^a^	π^b^	n^a^	π^b^	n^a^	π^2^	n^a^	π^b^
Humans	12	0.48±0.37	9	5.94±0.79	34	1.11±0.61	10	11.97±1.41
Livestock	4	0.34±0.37	3	0.51±0.28	10	0.40±0.39	4	12.75±1.47
Primates	1	—	4	5.34±0.74	9	1.08±0.60	1	—
All	17	0.65±0.41	16	6.63±0.81	53	1.14±0.56	15	14.17±1.431

a Sample sizes indicate numbers of individuals from which nucleotide sequences of the indicated locus were successfully generated.

b Genetic diversity is represented as percent nucleotide diversity (π; [46], ±standard error). Nucleotide diversity measures were calculated using the computer program MEGA4 [29], with standard errors estimated from 1,000 bootstrap resamplings of the sequence data.

**Figure 1:**
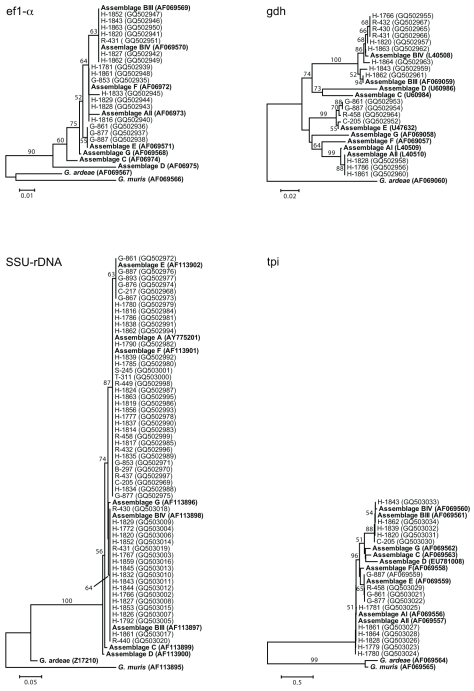
Dendrograms of *Giardia duodenalis* based on nucleotide sequences of four genes. Trees were constructed using the neighbor-joining method [47] and a Tamura-Nei/ maximum composite likelihood distance correction, implemented with the computer program MEGA4 [29]. Numbers above or below branches indicate bootstrap values (%), estimated from 1,000 resamplings of the sequence data; bootstrap values ≤50% are not shown. Taxon names indicate species (B = black-and-white colobus; C = cattle; G = goat; H = human; R = red colobus; S = sheep; T = red-tailed guenon), followed by identification numbers. GenBank accession numbers are shown in parentheses. Reference sequences are in bold. Numbers of taxa and identification numbers are different among trees because of different amplification success among loci. Scale bars indicate nucleotide substitutions per site.

Phylogenetic analyses of *G. duodenalis* nucleotide sequences yielded trees that were topologically similar across loci, but that differed in their phylogenetic resolution (Figure 1). The tree based on SSU-rDNA had the lowest phylogenetic resolution, but was nevertheless able to resolve *G. duodenalis* clades of assemblage E from livestock, assemblage A or F from humans, livestock, and primates, and assemblage B from humans and primates. The tree based on *tpi* was able to resolve *G. duodenalis* clades of assemblage B from humans and livestock, assemblage E from livestock and a red colobus, and assemblage A from humans. The tree based on *ef1-α* was able to resolve *G. duodenalis* clades of assemblage B from humans and primates and assemblage E from livestock, although the phylogenetic positions of other sequences remained ambiguous.

The tree based on *gdh* had the highest phylogenetic resolution and was able to resolve *G. duodenalis* clades of assemblage BIV from humans and red colobus, assemblage E from livestock and red colobus, and human-only clades of assemblage BIII and AII. In addition, the *gdh* tree was able to resolve genetic relationships among *G. duodenalis* sequences even within assemblages. Notably, *G. duodenalis* sequences from three red colobus and one human had identical *gdh* sequences within the assemblage BIV clade.

Using our phylogenetic results, we were able to classify 36 human *G. duodenalis* into either assemblage A or assemblage B. We were unable to detect an association between assemblage and any demographic, behavioral, or clinical variable, including age (≤15 years; OR = 0.32; 95% Wald confidence limits 0.05–1.96; Fisher's exact *P* = 0.200) or the reporting of gastrointestinal symptoms (OR = 1.60; 95% Wald confidence limits 0.33–7.85; Fisher's exact *P* = 0.430). We were, however, limited by a small sample size of infected children ≤5 years of age for whom both clinical data and *G. duodenalis* assemblage were available (n = 6); this precluded meaningful analysis of an association between assemblage and clinical disease in this age category, which might have been expected based on published results [30].

## Discussion

Our data provide evidence for multiple cross-species *G. duodenalis* transmission cycles in western Uganda. Our sequence analysis was able to resolve four *G. duodenalis* clades: one involving assemblage BIV in humans and primates, one involving assemblage E in livestock and primates, and two involving assemblages AII and BIII in humans. Our phylogeny based on *gdh* was additionally able to resolve sub-clades of *G. duodenalis* within assemblages, including from humans and red colobus within assemblage BIV and from livestock and red colobus within assemblage E. Although our small sample sizes precluded formal cladistic analyses of the directionality of cross-species transmission [31], we believe human-to-primate and livestock-to-primate transmission to be most likely in the case of assemblages BIV and E, respectively, given what is already known about the characteristic species that these assemblages infect [8]. Furthermore, the prevalence of *G. duodenalis* in humans was higher than in either primates or livestock (Table 1), as was the genetic diversity of *G. duodenalis* at 3 of 4 loci (Table 3), suggesting a human *G. duodenalis* reservoir for at least some assemblages.

The association of *G. duodenalis* assemblages AII, BIII, and BIV with humans and E with livestock as described above agrees with past research [8], [32], [33], as does our finding of assemblage B *G. duodenalis* in non-human primates, which has previously been documented in captive settings [34]. Our study adds new information about *G. duodenalis* assemblages in wild primates. Specifically, our finding of assemblage E *G. duodenalis* in red colobus is, to our knowledge, the first account this assemblage in a non-human primate. If assemblage E *G. duodenalis* can in fact be transmitted between livestock and primates, this would support findings by Foronda et al (2008), who inferred assemblage E *G. duodenalis* in another primate - humans - in Egypt from *tpi* gene sequences, implying a possible cattle-human transmission link [35]. Such “atypical” transmission might be enhanced in settings where ecological overlap between species is high, such as our study area, where primates must often cross cattle pastures to move between habitat patches [14]. In this light, we note that Graczyk et al (2002) analyzed 130 bp of the small subunit ribosomal RNA gene from *G. duodenalis* in people, cattle, and mountain gorillas in Bwindi Impenetrable National Park, Uganda (approximately 200 km from our study site), where ecological overlap between species is also high, and inferred infection with assemblage A in all three species [7]. That we did not find assemblage A *G. duodenalis* in species other than humans may reflect real differences between sites; however, we also note that SSU-rDNA had the lowest phylogenetic resolution of any gene in our study (Figure 1) and might thus be prone to causing errors of misclassification.

Our results also demonstrate that the prevalence of *G. duodenalis* in people in rural western Uganda is high, with up to approximately two thirds of people infected in some locations. These results are similar to those of other recent studies that have documented high rates of *G. duodenalis* infection in impoverished rural populations with limited access to health care, with prevalence ranging from a typical 20–30% to approximately 70% in some populations (e.g. [35]–[38]). Our prevalence estimates are considerably higher than has been reported for school children in Moyo District, Uganda (approximately 400 km from our study area) [39] or in the capital city of Kampala in school children [40] or children hospitalized with diarrhea [41], perhaps due to the higher sensitivity of our PCR-based detection method than the microscopic methods used in these studies. In this light, we note that the prevalence estimate we report here for primates (11.1%) is higher than in our previous investigation of these same primate populations [16], in which we documented a 3.8% *Giardia* prevalence using immunofluorescent microscopy (albeit with a separate set of samples collected two years earlier). This observation again suggests that PCR-based methods are more sensitive than microscopy.

We also documented a high local heterogeneity of *G. duodenalis* prevalence among human communities. People from Rurama community were infected with *G. duodenalis* at a rate approximately three times that of people in Bugembe or Kiko 1 communities, despite the roughly similar demographic composition of these communities and their close proximity to each other (within approximately 7 km [15]). Intriguingly, neither livestock nor primates in Rurama were infected with *G. duodenalis* at elevated rates, implying that general “parasite pollution” of the physical environment probably does not account for the trend observed in humans. We speculate that this variation may reflect local differences in human health or behavior, such as coinfection with other agents or methods of food preparation, respectively.

The lack of any measurable association between infection of people with *G. duodenalis* and the reporting of gastrointestinal symptoms was surprising, but it is concordant with some other recent studies. For example, Cordón et al. (2008) found *G. duodenalis* at 28.1% prevalence in diarrheic Peruvian children as well as in 19.5% of nondiarrheic children, emphasizing the importance of asymptomatic patients in *G. duodenalis* transmission where hygiene and sanitation are poor [42]. Other studies in India [24], Ethiopia [37], Bangladesh [38], and Peru [43] have found similarly weak or non-existent associations between *G. duodenalis* infection status and gastrointestinal symptoms.

Although we found little evidence for an effect of *G. duodenalis* on human health, we cannot exclude the possibility that the parasite may be impacting the health of livestock or that of wild primates. Such effects could, in turn, have consequences for conservation. The Ugandan red colobus monkey, for example, is endangered and may be declining due to a combination of nutritional stress and parasitism [44]. Increasing encroachment into primate habitats and consequent human-primate conflict ensure that, without intervention, such trends will continue [14].

We emphasize that our results are based small sample sizes and short nucleic acid sequences. Moreover, variable amplification success among loci and samples precluded phylogenetic analysis of concatenated sequences (*i.e.* multilocus sequence tying), which might have improved phylogenetic resolution. Findings such as our discovery of assemblage E *G. duodenalis* in red colobus monkeys, which would be the first report of this assemblage in a non-human primate, should ideally be confirmed with additional sampling and sequencing. Also, we were unable to examine the relationship between infection status and *Giardia*-specific treatment in people, which could have altered prevalence and the clinical manifestations of infection. We therefore view the results of this study as indicating a strong need for future research into the epidemiology, cross-species transmission ecology, and clinical consequences of *G. duodenalis* infection in both humans and wildlife, especially where these species interact in anthropogenically disturbed habitats in the tropics.
